# Role of Endoscopic Ultrasound-Guided Fine Needle Aspiration (EUS-FNA) in the Diagnosis of Suspicious Malignant Esophageal Strictures

**DOI:** 10.3390/jcm12062153

**Published:** 2023-03-09

**Authors:** Liang Min, Yumin Qing, Yi Chu, Chengbai Liang, Liang Lv, Deliang Liu, Yuyong Tan, Yuqian Zhou

**Affiliations:** 1 Department of Gastroenterology, The Second Xiangya Hospital, Central South University, Changsha 410011, China; 2 Research Center of Digestive Disease, Central South University, Changsha 410011, China

**Keywords:** esophageal strictures, endoscopic ultrasound-guided fine needle aspiration, rapid on-site evaluation

## Abstract

(1) Background: The accurate diagnosis of esophageal strictures is quite critical for optimizing medical intervention. However, the diagnosis of suspicious malignant esophageal strictures with intact mucosa appearance and negative biopsy results is challenging. This study aimed to evaluate the role of endoscopic ultrasound-guided fine needle aspiration (EUS-FNA) in the diagnosis of suspicious esophageal strictures. (2) Methods: We retrospectively analyzed the cases with suspicious malignant esophageal strictures that underwent EUS-FNA, with or without rapid on-site evaluation (ROSE), in our hospital from April 2017 to September 2022. Their clinical manifestations, imaging examinations, gastroscopic examinations, EUS-FNA results, and therapeutic strategies were retrospectively recorded and analyzed. (3) Results: A total of 23 patients (15 male and 8 female) were enrolled in this study. Based on EUS-FNA results, 18 patients were diagnosed with malignancies, including 16 cases of primary esophageal cancer (13 squamous carcinomas and 3 adenocarcinomas), 1 case of mediastinal cancer, and 1 case of metastatic esophageal cancer; 1 case of tuberculosis was also confirmed by EUS-FNA. Among 4 cases of ambiguous diagnosis with EUS-FNA, 1 was diagnosed with an esophageal glomus tumor after surgical removal, and 2 patients survived for several years without medical intervention, which hinted at the possibility of benign esophageal strictures. No major complications, including bleeding or perforation, were observed. (4) Conclusions: EUS-FNA may serve as a safe and effective diagnostic tool in suspicious malignant esophageal strictures with accurate specimen acquisition, especially for biopsy-negative cases.

## 1. Introduction

Esophageal stricture, which can be either benign or malignant, remains a commonly encountered clinical entity, resulting in dysphagia [1]. Benign strictures formation is the result of collagen and fibrous tissue deposition in patients with severe or chronic inflammation in the esophagus, while malignant strictures are caused by intrinsic luminal tumor growth or extrinsic esophageal compression [2]. Gastro-esophageal reflux disease (GERD); anastomotic, corrosive substance ingestion; eosinophilic esophagitis; and tuberculosis have been reported as the common causes of esophageal strictures [3,4]. However, extra caution should be taken when making diagnoses in clinical practice due to the possibility of malignant esophageal strictures. Generally, most malignancies can be pathologically diagnosed by gastroscopic biopsy. However, in some cases, the narrowed esophageal lumen makes it technically difficult for endoscopic evaluation and biopsy. Ultrathin endoscopy and a combination of endoscopy with dilation were implemented to provide a better view, as well as complete evaluation, even though there were some inevitable defects in these techniques [5,6]. Besides, some malignancies arising from the deep layer of the esophageal wall or adjacent organs may present with grossly intact appearances on gastroscopy. In these cases, the gastroscopic biopsy usually yields a negative result, as the biopsy forceps could not acquire the malignant tissue [7]. Even though positron emission tomography (PET)/computed tomography (CT), which could evaluate the lesions underneath the mucosa or from adjacent organs, could help in the diagnosis of esophageal lesions based on the imaging features of diseases [8,9], cytological and histological analysis remains the most reliable diagnostic criteria in esophageal strictures. Therefore, novel diagnostic methods which could penetrate the mucosa and reach those lesions are needed. In 1991, Vilmann first performed the ultrasound-guided fine needle aspiration (EUS-FNA) in a patient with cystadenoma in the head of the pancreas, which enabled the accurate histological and cytological confirmation of suspicious lesions [10]. This new technique was soon introduced in the diagnosis of various lesions of the gastrointestinal tract or adjacent organs, especially pancreatic lesions [11,12,13,14]. However, the reports of the application of EUS-FNA in the diagnosis of esophagus strictures are limited. This study aimed to evaluate the role of EUS-FNA, without esophageal dilation, in the diagnosis of suspicious malignant esophageal strictures with grossly intact mucosa appearance or negative biopsy. EUS-FNA could aid in the acquisition of the lesions, and further cytological or histological analysis would be a guide for diagnosis and treatment.

## 2. Materials and Methods

### 2.1. The Subjects

**Figure 1:**
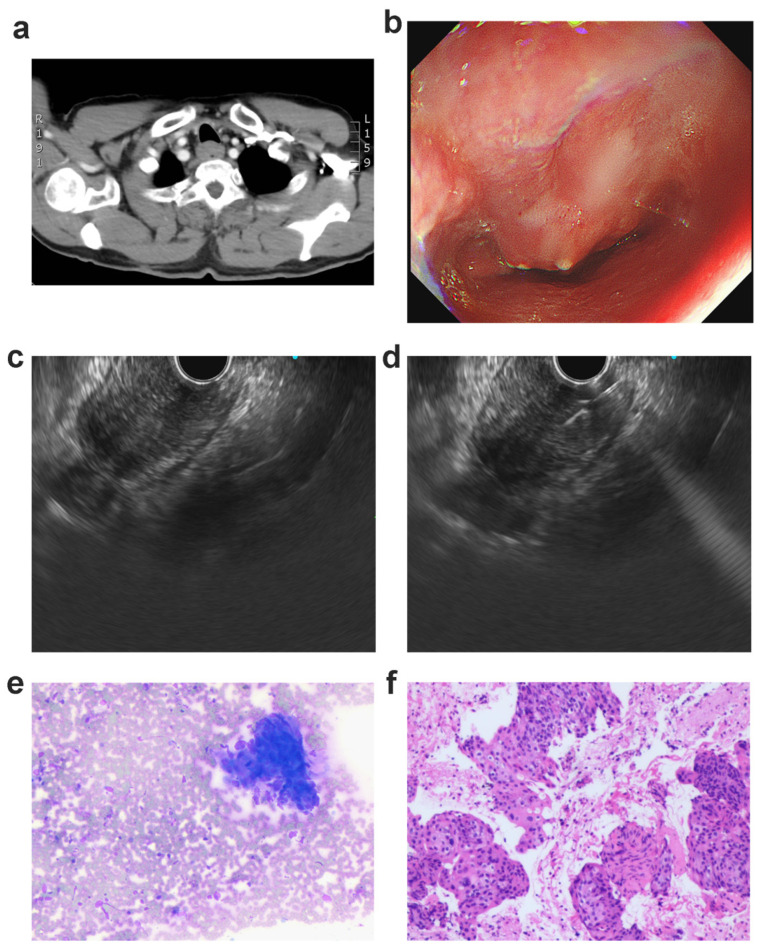
Medical information of patient 5: (**a**) thoracic computed tomography (CT) depicted the thickening of the esophageal wall at the thoracic entrance level; (**b**) endoscopy depicted esophageal stricture with mucosal erosion; (**c**) EUS (endoscopic ultrasound) depicted equal low-echo lesion at the upper esophagus; (**d**) EUS depicted needle puncture through the esophageal tissue; (**e**) rapid on-site evaluation depicted malignant cells; (**f**) histological studies with hematoxylin and eosin (H&E) staining showed malignant cells of poorly differentiated squamous cell carcinoma captured by EUS-FNA.

**Figure 2:**
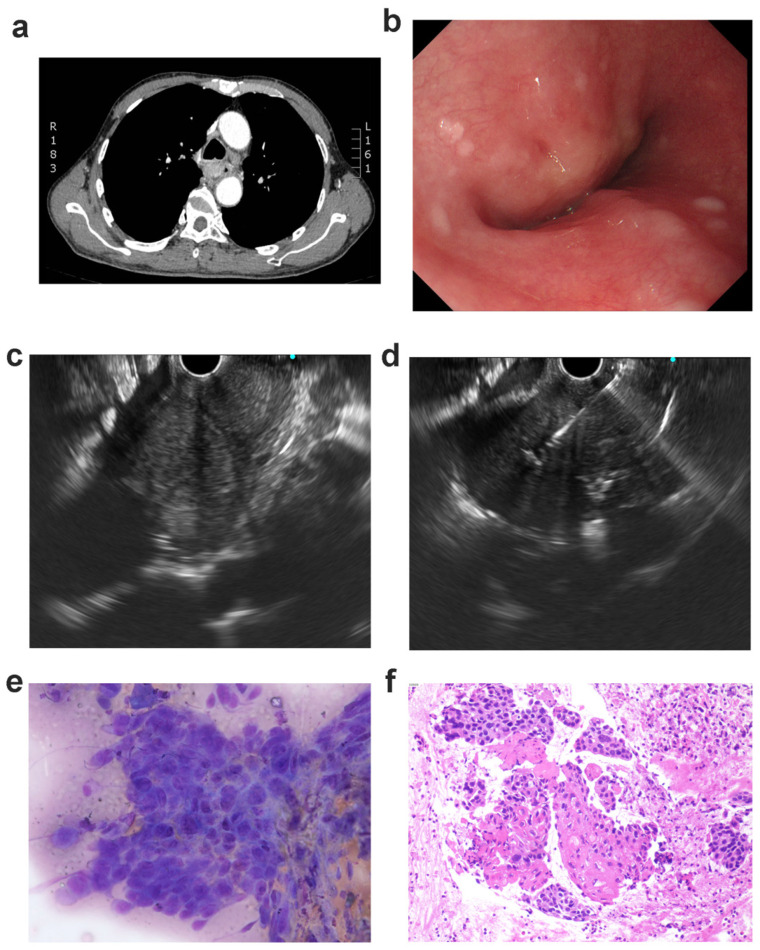
Medical information of patient 14: (**a**) thoracic computed tomography (CT) depicted the thickening of the esophageal wall at the middle esophagus; (**b**) endoscopy depicted esophageal stricture with intact mucosa; (**c**) EUS (endoscopic ultrasound) depicted low-echo lesion at the upper esophagus; (**d**) EUS depicted needle puncture through the esophageal tissue; (**e**) rapid on-site evaluation depicted malignant cells; (**f**) histological studies with hematoxylin and eosin (H&E) staining showed malignant cells of well-differentiated squamous cell carcinoma captured by EUS-FNA.

We retrospectively analyzed the patients with suspicious malignant esophageal strictures at the Department of Gastroenterology, the Second Xiangya Hospital of Central South University, from April 2017 to September 2022. A total of 23 patients who had a suspicious malignant esophageal stricture and underwent EUS-FNA were enrolled in our retrospective study. All patients presented with various degrees of dysphagia or weight loss, and thoracic CT demonstrated thickness of the esophageal wall, compression of the esophageal wall, lymphadenopathy, or mediastinal mass. Esophageal strictures were confirmed by gastroscopy, and typic mucosa alterations of malignancy were absent in all patients. EUS-FNA, with or without rapid on-site evaluation (ROSE), was conducted to obtain a histopathological diagnosis. The clinical manifestations, imaging examinations, gastroscopic examinations, and EUS-FNA results were recorded and analyzed (Figure 1 and Figure 2). This study was conducted in accordance with the Declaration of Helsinki, and was approved by the ethics committee of our hospital. All patients were informed of the potential risk of EUS-FNA and signed the informed consent form.

### 2.2. Materials

An electronic curved linear array ultrasound gastroscope (GF-UCT260, Olympus, Tokyo, Japan) was used for lesion orientation. A 19-G (Expect Slimline, Boston Scientific, Marlborough, MA, USA), a 22-G (Expect Slimline, Boston Scientific, Marlborough, MA, USA), a 22-G (Chiba biopsy needle, Cook Medical, Bloomington, IN, USA), a 22-G (SonoTip TopGain, Medi-Global, Achenmuehle, Germany), a 25-G (Expect Slimline, Boston Scientific, Marlborough, MA, USA), or a 25-G (SonoTip TopGain, Medi-Global, Achenmuehle, Germany) was used for puncture. The puncture needles were chosen by the operator, based on the size, site, and blood supply of the lesions.

### 2.3. EUS-FNA Protocol

Preoperative routine examinations, including blood routine, coagulation function, electrocardiogram (ECG), and thoracic CT, were performed to rule out potential contraindications, such as severe cardiopulmonary diseases and coagulation disorders. Intramuscular injection of diazepam and bucinnazine hydrochloride was performed before the operation in all patients to provide sedation and analgesia. Esophageal strictures were classified based on their location along the esophagus as the distance from the incisors, as previously described: upper esophageal strictures were defined as those 15–23 cm from the incisors, middle esophageal strictures as those 24–32 cm from the incisors, and lower esophageal strictures as those >32 cm from the incisors [15]. The procedure was conducted by three experienced endoscopists (Yi Chu, Yuyong Tan, and Yuqian Zhou), and a visual inspection was performed to confirm the characteristics of the lesions, which could help determine the optimal device and puncture path. Ultrasound scanning was then applied, and a needle was inserted with ultrasound guidance. The stylet was pulled out immediately when the needle tip was in the appropriate position. Later on, a negative pressure syringe with 5–10 mL pressure was connected, and the needle was moved back and forth within the lesion. All patients were observed for 12 h post-procedure for any potential complications.

### 2.4. Sample Processing

Smears were prepared for rapid on-site evaluation (ROSE), if necessary. Alcohol-fixed slides and tissue blocks were prepared with bloody components of suction and small tissue within the needle for further analysis. Hematoxylin and eosin (H&E) staining was preferred for the cytological and histological analysis, and immunohistochemical (IHC) staining was also performed, when necessary.

### 2.5. Therapeutic Strategies and Follow-Up

The hospital’s electronic medical record system was searched to follow subsequent treatment. For patients without a definite diagnosis, telephone follow-up was conducted to check the potential diagnosis and patients’ status. Telephone follow-up was also implemented for patients who declined medical intervention.

### 2.6. Statistical Analysis

IBM SPSS Statistics (Version 26.0, IBM, Armonk, NY, USA) was used for data analysis. Continuous variables were presented as mean with median and range, and categorical variables as the frequency with percentage.

## 3. Results

A total of 23 patients (15 male and 8 female) were enrolled in our study, and the average age was 62.6 years (median = 62, range = 45–81). All patients presented various degrees of dysphagia, and the average duration of the symptom was 6.3 months (median = 3, range = 0.7–24). Additionally, body weight loss was reported in 11 patients (47.8%). A Thoracic CT scan was performed in all patients, and the thoracic CT scan revealed thickening of the esophageal wall in 19 cases (82.6%), lymphadenopathy in 14 cases (60.9%), and mediastinal lesions in 4 cases (17.4%). Endoscopic biopsies were performed on 20 patients (87.0%), and 3 of them (13.0%) received repeated biopsies before EUS-FNA, all of which were negative (Table 1).

Based on the endoscopic evaluation, the esophageal strictures were located in the upper esophagus in 5 patients (21.7%), the middle esophagus in 11 patients (47.8%), and the lower esophagus in 7 patients (30.4%). Endoscopic examination revealed rough mucosa in 6 patients (26.1%), mucosal erosion in 3 patients (13.0%), and smooth stricture in 14 patients (60.9%). The endoscopy was not able to pass through the stricture in 12 patients (52.2%). For puncture, 19-G needles were used in 2 cases (8.7%), 22-G needles in 17 cases (73.9%), and 25-G needles in 4 cases (17.4%). The EUS-FNA biopsy was performed in all patients, detecting malignancy in 18 patients (78.3%), tuberculosis in 1 patient (4.3%), and undefined lesions in 4 patients (17.4%) by cytological and histological study. Among the 4 patients with undefined lesions, 1 patient underwent surgery and was diagnosed with an esophageal glomus tumor, and 2 of them, who were followed for 44 months and 24 months, respectively, were alive at the last follow-up in January 2023, while the other was lost to follow-up (Supplementary Materials Table S1).

Among those patients with malignant suspicious esophageal stricture, esophageal cancer was the most common cause, as 16 patients were diagnosed of primary esophageal cancer (13 esophageal squamous cell carcinomas and 3 esophageal adenocarcinomas). There was also mediastinal malignancy in 1 patient, and recurrent lung adenoid cystic carcinoma with esophagus metastasis in 1 patient. No obvious complications, including infection, bleeding, esophageal fistula, or perforation, were observed during hospitalization. Among the 18 patients who were diagnosed with malignancy, ROSE was conducted in 12 patients, cytological study in 15 patients, and histological study in 18 patients. ROSE found malignant cells in 11 cases, yielding a positive rate of 91.7%, while cytological studies yielded a positive rate of 60% (9/15). Histological studies were positive in all 18 patients diagnosed with malignancy (Table 2).

Among the 16 patients with primary esophageal cancer, 4 cases were treated with surgery, 1 case with chemotherapy followed by surgery, 1 case with chemotherapy, 2 cases with chemoradiotherapy, 1 with immunotherapy, 4 with stent placement, and 3 patients declined treatment. Chemotherapy was adapted in the patient with mediastinal malignancy, and a combination of chemotherapy and immunotherapy was used for metastatic esophageal cancer. As there were 3 patients with malignancies who declined further medical intervention, we performed telephone follow-ups in those patients. Among those patients, 2 of them died 5 months and 13 months later, respectively, and the other was still alive after 11 months of follow-up.

## 4. Discussion

Esophageal strictures could be caused by various pathogenic processes, including benign disease, malignancy, and endoscopic intervention [16]. It is of vital importance to make the correct diagnosis in order to choose the appropriate management, especially for suspicious malignancies. The narrowed esophageal lumen usually impeded optic evaluation and precluded the passage of a gastroscope. As a combination of endoscopy, biopsy, and dilation was introduced in the 1980s; esophageal dilation which could provide a wide view for gastroenterologists was utilized in the evaluation procedure of esophageal strictures [17]. However, there were concerns about severe complications, including perforation [18]; particularly in patients with esophageal cancer, the perforation rate was up to 10.6% [19]. Mulcahy reports the use of the ultrathin endoscope, which is less than 6mm in diameter and could pass through some of the strictures that could not be penetrated by an ordinary gastroscope, in the assessment of gastrointestinal strictures [20]. However, the ultrathin endoscopy could not guarantee the successful endoscopic evaluation in all patients with gastrointestinal strictures. The ultrathin endoscope is not sufficiently solid for manipulatation due to the smaller diameter, and the small work channel may impede the tissue acquisition [6,21]. Besides, there are esophageal strictures caused by submucosa or extrinsic lesions exhibiting grossly intact mucosa, which would impede the accurate acquisition of target lesions by gastroscopic biopsy and potentially lead to misdiagnoses. Thus, diagnostic methods that could evaluate the lesions underneath the mucosa or adjacent organs were implemented. In 1998, Faugel reported 4 biopsy-negative malignant esophageal strictures diagnosed by EUS, which demonstrated that EUS could be a useful tool in the evaluation of suspicious esophageal strictures [22]. Nevertheless, there are some defects of EUS in clinical practice: Firstly, in some cases, inflammation might lead to the loss of the normal sonographic appearance, which could be confused with early-stage cancer; secondly, EUS is not applicable when the endoscope fails to pass through the lumen, and esophageal dilation will be needed; Thirdly, EUS could not provide histopathological results which could help optimize the treatment and management of the condition. Similarly, PET/CT has limited utility in the diagnosis of suspicious esophageal strictures due to the inability to provide histopathological results; therefore, it could serve as a supplementary tool. EUS-FNA, which was firstly introduced in the 1990s, could overcome the defects of EUS, as a needle is used to acquire target lesions with ultrasound guidance. EUS-FNA has been an established and highly accurate method for esophageal cancer staging, but there was no consensus on the application of EUS-FNA in the diagnosis of esophageal strictures [23].

Currently, only a limited number of studies have reported the role of EUS-FNA in patients with esophageal strictures [7,24,25,26]. In 2013, Adrián-de-Ganzo reported the 2 cases of linitis-like squamous esophageal cancer diagnosed by EUS-FNA after esophageal dilation, which was the first reported application of EUS-FNA in the diagnosis of biopsy-negative malignant esophageal strictures [24]. Due to the concern about the serious complications of esophageal dilation, EUS-FNA without dilation was implemented in later practice. Dahale demonstrated 11 smooth esophageal strictures caused by esophageal cancer, which were diagnosed by EUS-FNA, without esophageal dilation, in 2019 [7]. Pan reported one case of biopsy-negative intramural esophageal squamous cell carcinoma, without mucosa invasion, which was diagnosed by EUS-FNA, without esophageal dilation, in 2020 [25]. Cao conducted a cross-sectional study containing 50 benign-appearing malignant esophageal strictures in 2022, which demonstrated the effectiveness and safety of EUS-FNA, without esophageal dilation, in the diagnosis of malignant esophageal strictures [26]. These 4 studies reported the application of EUS-FNA in the diagnosis of malignant esophageal strictures, whereas the accurate diagnosis of suspicious esophageal strictures, which could be either benign or malignant, was a huge challenge for gastroenterologists in clinical practice. Suspicious malignant esophageal strictures, which generally presented with lymphadenopathy or body weight loss, might cause concerns about malignancy among patients and doctors. Hence, accurate diagnoses are of vital importance for those patients with suspicious malignant esophageal stricture to optimized the management and therapeutic strategies. While a limited number of studies demonstrated the application of EUS-FNA in the diagnosis of malignant esophageal strictures, there were no reports on distinguishing suspicious esophageal strictures by EUS-FNA.

Rapid on-site evaluation (ROSE), which is intended to provide the real-time evaluation of sample adequacy and diagnostic yield, remains one of the most controversial topics in the field of EUS-FNA [27]. Currently, EUS-FNA with ROSE is mainly applied in pancreatic lesions. While studies reported the improvement of diagnostic performance and reduction of needle passes in EUS-FNA with ROSE [28,29], there were doubts about the benefits of ROSE [30,31]. The European Society of Gastrointestinal Endoscopy (ESGE) Technical Guidelines equally recommend the EUS-FNA, with or without ROSE [32], and the Korean Society of Gastrointestinal Endoscopy (KESG) suggested that the routine application of ROSE cannot guarantee an improvement in diagnostic accuracy and performance of EUS-FNA [33]. However, there were no reports of the application of ROSE in the diagnosis of suspicious malignant esophageal strictures.

In the present study, 23 cases with suspicious esophageal strictures were enrolled, and EUS-FNA without dilation was introduced. Thoracic CT scanning demonstrated the thickness of the esophageal wall, compression of the esophageal wall, adenopathy, or mediastinal mass in all patients. A total of 18 cases of malignancies were diagnosed by EUS-FNA, including 16 cases of primary esophageal cancer, 1 case of metastatic esophageal cancer, and 1 case of mediastinal cancer. The occurrence of 1 benign lesion of tuberculosis was also confirmed. However, there were 4 cases that could not be accurately diagnosed by EUS-FNA, among which 1 patient was diagnosed with a glomus tumor after surgery, whereas 3 patients refused further examination. The ambiguous diagnoses might be caused by a lack of target tissues, which could potentially be solved by repeat sampling. While the accurate diagnosis of suspicious esophageal strictures was extremely difficult, many patients had spent huge amounts of time and money and were unwilling to submit to further examination and intervention. Therefore, it is quite important to apply EUS-FNA during the early diagnostic process and to improve puncture accuracy. Among 3 patients refusing further examination and intervention, 2 of them survived for several years without an obvious deterioration of health, which highly indicated the possibility of benign esophageal strictures. While malignancies could be confirmed by cancer cells depicted by ROSE, histological, or histological studies, it is quite difficult to draw a conclusion of benign lesions due to the lack of a specific marker and the concern of inaccurate sampling or processing, which was a common dilemma for biopsy, which only obtains a small piece of tissue for pathologists to analysis. To improve the diagnosis accuracy, in this study, ROSE was introduced in combination with cytological and histological studies. In 12 cases with malignant esophageal strictures, ROSE depicted cancer cells in 11 cases, which demonstrated the potential application of ROSE as a supplementary method. While EUS-FNA, combined with ROSE, demonstrated non-inferior performance compared to EUS-FNA without ROSE, the major obstacles to the wide application of ROSE include limiting pathologist staffing, and a longer procedures time [27]. It should be addressed that in patient 11, even though both cytological and histological studies reported suspicious round tumor cells, with little cytoplasm, while no malignant cell was reported by ROSE, the final diagnosis was an esophageal glomus tumor, which is an extremely rare esophageal neoplasm [34].

In patients diagnosed with malignancy, only 7 patients underwent curative-intended treatment, including surgery in 4 patients, chemotherapy followed by surgery in 1 patient, and chemoradiotherapy in 2 patients. The majority of patients underwent palliative therapy or gave up, as the malignancies were at late stages when they were confirmed. On one hand, patients with suspicious malignant esophageal strictures usually exhibited no obvious symptoms at the early stages of cancers, as dysphagia occurred when the strictures obstructed 50% of esophageal lumen [35], while on the other hand, traditional methods, such as gastroscopic biopsy yielding negative results, could halt timely medical intervention, and the application of EUS-FNA on a large-scale could potentially aid diagnosis at early stages.

This study demonstrated the potential application of EUS-FNA, with or without ROSE, in the diagnosis of suspicious esophageal strictures. While it has previously been a well-established tool for esophageal cancer staging, EUS-FNA could also be a promising diagnostic method for suspicious malignant esophageal strictures. However, our study had a few limitations: firstly, this is a retrospective study with a relatively small sample size, with only 23 patients included, with ambiguous diagnoses in 3 patients without further confirmed diagnoses; secondly, no comparison was conducted on the diagnostic yield between EUS-FNA and other diagnostic methods, such as endoscopic mucosal resection or surgical biopsy; thirdly, we did not provide information about the cost-effectiveness of EUS-FNA compared to other diagnostic methods, as EUS-FNA may be more expensive or less readily available in some clinical settings, although it may be less invasive than other approaches. Thus, prospective, multiple-center, comparative studies with larger sample sizes are needed to verify the diagnostic role of EUS-FNA in esophageal stricture and to optimize the procedure.

## 5. Conclusions

EUS-FNA, with or without ROSE, which does not require esophageal dilation, could provide accurate specimen acquisition in suspicious malignant esophageal strictures caused by various pathogenic factors. Definite diagnoses could be made in most cases, based on cytological and histological studies. For suspicious malignant esophageal strictures with intact mucosa appearance or negative biopsy results, EUS-FNA could be the optimal diagnostic tool.

## Figures and Tables

**Table 1:** General characteristics of patients.

Patient ID	Sex	Age	Symptoms		Thoracic CT			Number of Endoscopic Biopsy before EUS-FNA
			Duration of Dysphagia (month)	Body Weight Loss (Kg)	Thickening of Esophageal Wall	Mediastinal Mass	Lymphadenopathy	
Patient 1	F	54	6.0	NA	✓		✓	1
Patient 2	M	68	6.0	1.5	✓		✓	1
Patient 3	F	69	3.0	5.0	✓		✓	1
Patient 4	M	70	2.0	2.0	✓			1
Patient 5	M	59	24.0	7.5	✓		✓	1
Patient 6	F	45	12.0	NA	✓		✓	1
Patient 7	F	76	24.0	3.0			✓	1
Patient 8	F	65	1.0	NA	✓		✓	1
Patient 9	F	52	8.0	NA		✓		1
Patient 10	M	81	12.0	4.0	✓	✓		1
Patient 11	F	61	0.7	NA	✓			1
Patient 12	M	52	3.0	NA	✓		✓	2
Patient 13	M	53	3.0	NA	✓		✓	1
Patient 14	M	66	1.0	NA	✓		✓	0
Patient 15	M	61	1.0	5.0		✓		1
Patient 16	M	48	2.0	NA	✓		✓	1
Patient 17	M	66	1.0	5.0		✓		0
Patient 18	M	73	24.0	6.5	✓			2
Patient 19	M	62	2.0	20.0	✓		✓	1
Patient 20	M	54	1.0	NA	✓			1
Patient 21	M	77	3.0	NA	✓		✓	1
Patient 22	M	54	3.0	5.0	✓			2
Patient 23	F	74	2.0	NA	✓		✓	0

Abbreviation: F: female; M: male; “NA” represents no obvious body weight loss reported by the patient; “✓” represents the existence of corresponding features.

**Table 2:** EUS-FNA.

Patient ID	Endoscopy				Malignant Cell Positive			Diagnosis
	Strictures Location	Endoscope Pass	Mucosa Appearance	Needle	ROSE	Cytology	Histology	
Patient 1	U	✓	Intact	25G	(-)	(-)	(-)	Undefined
Patient 2	U		Rough	22G	NA	(-)	(+)	ESCC
Patient 3	U		Intact	22G	(+)	(+)	(+)	ESCC
Patient 4	U	✓	Intact	22G	(+)	(+)	(+)	ESCC
Patient 5	U		Erosion	22G	(+)	(+)	(+)	ESCC
Patient 6	M		Intact	22G	(-)	(-)	(-)	Undefined
Patient 7	M	✓	Rough	22G	NA	NA	(-)	Undefined
Patient 8	M	✓	Erosion	25G	NA	(-)	(-)	TB
Patient 9	M		Intact	22G	(-)	(-)	(+)	Metastatic esophageal cancer *
Patient 10	M		Intact	22G	(+)	(-)	(+)	EAC
Patient 11	M	✓	Intact	22G	(-)	(+)	(+)	Esophageal glomus tumor *
Patient 12	M		Intact	22G	(+)	NA	(+)	ESCC
Patient 13	M		Rough	22G	NA	(+)	(+)	ESCC
Patient 14	M		Intact	25G	(+)	(+)	(+)	ESCC
Patient 15	M	✓	Rough	22G	NA	NA	(+)	ESCC
Patient 16	M		Rough	22G	(+)	(+)	(+)	ESCC
Patient 17	L	✓	Intact	22G	(+)	(+)	(+)	Mediastinal malignancy
Patient 18	L	✓	Intact	19G	(+)	(-)	(+)	EAC
Patient 19	L		Intact	25G	NA	(-)	(+)	EAC
Patient 20	L	✓	Intact	19G	NA	NA	(+)	ESCC
Patient 21	L	✓	Rough	22G	NA	(+)	(+)	ESCC
Patient 22	L		Erosion	22G	(+)	(+)	(+)	ESCC
Patient 23	L	✓	Intact	22G	(+)	(-)	(+)	ESCC

Abbreviation: U: upper esophagus; M: middle esophagus; L: lower esophagus; ROSE: rapid on-site evaluation; NA: ROSE or cytological study was not performed; ESCC: esophageal squamous cell carcinoma; EAC: esophageal adenocarcinoma; TB: tuberculosis. Metastatic esophageal cancer *: recurrent lung adenoid cystic carcinoma with esophagus metastasis; Esophageal glomus tumor *: diagnosed by surgical removal.

## Data Availability

The data presented in this study are available on request from the corresponding author.

## Supplementary Materials

The following supporting information can be downloaded at: https://www.mdpi.com/article/10.3390/jcm12062153/s1, Table S1: Treatment and follow-up.

## References

[1] Siersema P.D. (2008). Treatment Options for Esophageal Strictures. Nat. Rev. Gastroenterol. Hepatol..

[2] Pasha S.F., Acosta R.D., Chandrasekhara V., Chathadi K.V., Decker G.A., Early D.S., Evans J.A., Fanelli R.D., Fisher D.A., ASGE Standards of Practice Committee (2014). The Role of Endoscopy in the Evaluation and Management of Dysphagia. Gastrointest. Endosc..

[3] Desai J.P., Moustarah F. (2022). Esophageal Stricture. StatPearls.

[4] Ruigómez A., Alberto García Rodríguez L., Wallander M.-A., Johansson S., Eklund S. (2006). Esophageal Stricture: Incidence, Treatment Patterns, and Recurrence Rate. Off. J. Am. Coll. Gastroenterol. ACG.

[5] Bryant A.S., Cerfolio R.J. (2007). Esophageal Trauma. Thorac. Surg. Clin..

[6] Tanuma T., Morita Y., Doyama H. (2016). Current Status of Transnasal Endoscopy Worldwide Using Ultrathin Videoscope for Upper Gastrointestinal Tract. Dig. Endosc..

[7] Dahale A.S., Srivastava S., Sonika U., Dalal A., Goyal A., Sakhuja P., Sachdeva S., Puri A.S. (2020). Role of Linear Endosonography in the Diagnosis of Biopsy-Negative Malignant Esophageal Strictures: Exploring the Unexplored. JGH Open.

[8] Rice T.W. (2000). Clinical Staging of Esophageal Carcinoma. CT, EUS, and PET. Chest Surg. Clin. N. Am..

[9] Ott K., Weber W., Siewert J.-R. (2006). The Importance of PET in the Diagnosis and Response Evaluation of Esophageal Cancer. Dis. Esophagus.

[10] Vilmann P., Jacobsen G.K., Henriksen F.W., Hancke S. (1992). Endoscopic Ultrasonography with Guided Fine Needle Aspiration Biopsy in Pancreatic Disease. Gastrointest. Endosc..

[11] Levy M.J., Abu Dayyeh B.K., Fujii L.L., Clayton A.C., Reynolds J.P., Lopes T.L., Rao A.S., Clain J.E., Gleeson F.C., Iyer P.G. (2015). Detection of Peritoneal Carcinomatosis by EUS Fine-Needle Aspiration: Impact on Staging and Resectability (with Videos). Gastrointest. Endosc..

[12] Vilmann P., Hancke S., Henriksen F.W., Jacobsen G.K. (1993). Endosonographically-Guided Fine Needle Aspiration Biopsy of Malignant Lesions in the Upper Gastrointestinal Tract. Endoscopy.

[13] Silvestri G.A., Hoffman B.J., Bhutani M.S., Hawes R.H., Coppage L., Sanders-Cliette A., Reed C.E. (1996). Endoscopic Ultrasound with Fine-Needle Aspiration in the Diagnosis and Staging of Lung Cancer. Ann. Thorac. Surg..

[14] Singh P., Mukhopadhyay P., Bhatt B., Patel T., Kiss A., Gupta R., Bhat S., Erickson R.A. (2009). Endoscopic Ultrasound versus CT Scan for Detection of the Metastases to the Liver: Results of a Prospective Comparative Study. J. Clin. Gastroenterol..

[15] Tan Y., Lv L., Duan T., Zhou J., Peng D., Tang Y., Liu D. (2016). Comparison between Submucosal Tunneling Endoscopic Resection and Video-Assisted Thoracoscopic Surgery for Large Esophageal Leiomyoma Originating from the Muscularis Propria Layer. Surg. Endosc..

[16] Adler D.G., Siddiqui A.A. (2017). Endoscopic Management of Esophageal Strictures. Gastrointest. Endosc..

[17] Barkin J.S., Taub S., Rogers A.I. (1981). The Safety of Combined Endoscopy, Biopsy and Dilation in Esophageal Strictures. Am. J. Gastroenterol..

[18] Hagel A.F., Naegel A., Dauth W., Matzel K., Kessler H.P., Farnbacher M.J., Hohenberger W.M., Neurath M.F., Raithel M. (2013). Perforation during Esophageal Dilatation: A 10-Year Experience. J. Gastrointest. Liver Dis..

[19] Molina J.C., Goudie E., Pollock C., Menezes V., Ferraro P., Lafontaine E., Martin J., Nasir B., Liberman M. (2021). Balloon Dilation for Endosonographic Staging in Esophageal Cancer: A Phase 1 Clinical Trial. Ann. Thorac. Surg..

[20] Mulcahy H.E., Fairclough P.D. (1998). Ultrathin Endoscopy in the Assessment and Treatment of Upper and Lower Gastrointestinal Tract Strictures. Gastrointest. Endosc..

[21] Aydinli M., Koruk I., Dag M.S., Savas M.C., Kadayifci A. (2012). Ultrathin Endoscopy for Gastrointestinal Strictures. Dig. Endosc..

[22] Faigel D.O., Deveney C., Phillips D., Fennerty M.B. (1998). Biopsy-Negative Malignant Esophageal Stricture: Diagnosis by Endoscopic Ultrasound. Am. J. Gastroenterol..

[23] Maple J.T., Peifer K.J., Edmundowicz S.A., Early D.S., Meyers B.F., Jonnalagadda S., Azar R.R. (2008). The Impact of Endoscopic Ultrasonography with Fine Needle Aspiration (EUS-FNA) on Esophageal Cancer Staging: A Survey of Thoracic Surgeons and Gastroenterologists. Dis. Esophagus.

[24] Adrián-de-Ganzo Z., Gimeno-García A.Z., Elwassief A., Paquin S., Sahai A.V., García-Castro C., Nicolás-Pérez D., Brito-García A., Martín-Corriente M.D.C., Garièpy G. (2013). Linitis-like Squamous Esophageal Cancer Diagnosed by Endoscopic Ultrasonography-Guided Fine-Needle Aspiration Cytology: Report of Two Cases. Eur. J. Gastroenterol. Hepatol..

[25] Pan H., Zhou X., Zhao F., Lou G. (2020). The Diagnosis of Intramural Esophageal Squamous Cell Carcinoma without Mucosal Invasion Using Endoscopic Ultrasound-Guided Fine Needle Aspiration Biopsy. Medicine.

[26] Cao F., Chen G., Su W., Zhang Z., Fu Q., Zhou D., Dai Z. (2022). Endoscopic Ultrasound-Guided Fine Needle Aspiration for Smooth Benign Appearing Malignant Esophageal Stricture: A Cross-Sectional Study. J. Thorac Dis.

[27] Yang F., Liu E., Sun S. (2019). Rapid On-Site Evaluation (ROSE) with EUS-FNA: The ROSE Looks Beautiful. Endosc. Ultrasound.

[28] Hébert-Magee S., Bae S., Varadarajulu S., Ramesh J., Frost A.R., Eloubeidi M.A., Eltoum I.A. (2013). The Presence of a Cytopathologist Increases the Diagnostic Accuracy of Endoscopic Ultrasound-Guided Fine Needle Aspiration Cytology for Pancreatic Adenocarcinoma: A Meta-Analysis. Cytopathology.

[29] Haba S., Yamao K., Bhatia V., Mizuno N., Hara K., Hijioka S., Imaoka H., Niwa Y., Tajika M., Kondo S. (2013). Diagnostic Ability and Factors Affecting Accuracy of Endoscopic Ultrasound-Guided Fine Needle Aspiration for Pancreatic Solid Lesions: Japanese Large Single Center Experience. J. Gastroenterol..

[30] Kong F., Zhu J., Kong X., Sun T., Deng X., Du Y., Li Z. (2016). Rapid On-Site Evaluation Does Not Improve Endoscopic Ultrasound-Guided Fine Needle Aspiration Adequacy in Pancreatic Masses: A Meta-Analysis and Systematic Review. PLoS ONE.

[31] Guvendir I., Zemheri I.E., Ozdil K. (2022). Impact of Rapid On-Site Evaluation on Diagnostic Accuracy of EUS-Guided Fine-Needle Aspiration of Solid Pancreatic Lesions: Experience from a Single Center. BMC Gastroenterol..

[32] Polkowski M., Jenssen C., Kaye P., Carrara S., Deprez P., Gines A., Fernández-Esparrach G., Eisendrath P., Aithal G.P., Arcidiacono P. (2017). Technical Aspects of Endoscopic Ultrasound (EUS)-Guided Sampling in Gastroenterology: European Society of Gastrointestinal Endoscopy (ESGE) Technical Guideline—March 2017. Endoscopy.

[33] Chung M.J., Park S.W., Kim S.-H., Cho C.M., Choi J.-H., Choi E.K., Lee T.H., Cho E., Lee J.K., Song T.J. (2021). Clinical and Technical Guideline for Endoscopic Ultrasound (EUS)-Guided Tissue Acquisition of Pancreatic Solid Tumor: Korean Society of Gastrointestinal Endoscopy (KSGE). Gut Liver.

[34] Nishida K., Watanabe M., Yamamoto H., Yoshida R., Fujita A., Koga T., Kajiyama K. (2013). Glomus Tumor of the Esophagus. Esophagus.

[35] de Wijkerslooth L.R.H., Vleggaar F.P., Siersema P.D. (2011). Endoscopic Management of Difficult or Recurrent Esophageal Strictures. Off. J. Am. Coll. Gastroenterol. ACG.

