# Supplementary material for: Role of Endoscopic Ultrasound-Guided Fine Needle Aspiration (EUS-FNA) in the Diagnosis of Suspicious Malignant Esophageal Strictures
Source: J Clin Med. 2023 Mar 9;12(6):2153. doi: 10.3390/jcm12062153 (PMC10057162; doi:10.3390/jcm12062153)
Supplement: Supplementary file 1 [file jcm-12-02153-s001.zip › jcm-2247977-supplementary.pdf]

Table S1. General characteristics of patients

Patient ID	Treatment									Follow-up	
	Surgery	Chemotherapy + Surgery	Chemotherapy	Chemotherapy + radiotherapy	Chemotherapy + Immunotherapy	Immunotherapy	Stent placememt	Anti-TB treatment	Give-up	Duration (month)	Status
Patient 1									✓	44	Alive
Patient 2							✓				
Patient 3							✓				
Patient 4							✓				
Patient 5				✓							
Patient 6									✓	24	Alive
Patient 7										Lost to follow-up	
Patient 8								✓			
Patient 9			✓								
Patient 10							✓				
Patient 11	✓										
Patient 12						✓					
Patient 13	✓										
Patient 14									✓	13	Dead
Patient 15				✓							
Patient 16		✓									
Patient 17					✓						
Patient 18									✓	5	Dead
Patient 19	✓										
Patient 20			✓								
Patient 21	✓										
Patient 22	✓										
Patient 23									✓	12	Alive

"Anti-TB treatment" represents anti-tuberculosis treatment; "✓" represents the corresponding therapies were applied.
